# Trousseau’s Syndrome With Severe Visual Loss As the Initial Symptom

**DOI:** 10.7759/cureus.56211

**Published:** 2024-03-15

**Authors:** Atsushi Okubo, Ryo Obata, Keiko Azuma, Shuichi Kishimoto, Mikiro Mori

**Affiliations:** 1 Ophthalmology, Saitama Red Cross Hospital, Saitama, JPN; 2 Ophthalmology, University of Tokyo, Tokyo, JPN; 3 Ophthalmology, Toranomon Hospital, Tokyo, JPN

**Keywords:** disseminated intravascular coagulation, vision loss, malignant tumors, hypercoagulability, trousseau syndrome

## Abstract

There are limited reports on patients with Trousseau syndrome, a condition characterized by hypercoagulability associated with malignant tumors, initially manifesting with reduced visual function. We present a case of a patient who experienced bilateral vision loss and was subsequently diagnosed with Trousseau's syndrome following examination and investigations. A 70-year-old man, undergoing chemotherapy for advanced pancreatic cancer, reported decreased visual acuity in both eyes. A dilated fundus examination revealed retinal pigment epithelial atrophy in the posterior pole and cotton-wool spots. Optical coherence tomography exhibited partial disruption of the ellipsoid zone in the parafoveal region, and full-field electroretinogram results were subnormal, although the macular retinal structure was preserved. Brain magnetic resonance imaging (MRI) detected occipital lobe infarction. Elevated coagulability markers, including D-dimer (5.5μg/mL), led to the diagnosis of Trousseau's syndrome. In cases where patients with malignant tumors present with profound visual loss, considering the possibility of Trousseau's syndrome and conducting assessments of brain function and coagulability is crucial for accurate diagnosis and appropriate management.

## Introduction

Trousseau's syndrome is defined as a hypercoagulable state or disseminated intravascular coagulation (DIC) associated with malignant tumors [1]. The pathogenesis of this disease is complex and not fully understood, but it is believed that the central mechanisms involve thrombosis due to DIC associated with adenocarcinoma or cardiogenic embolism caused by nonbacterial thromboendocarditis (NBTE) [1]. Additionally, it is not uncommon for malignant tumors to be discovered following the onset of cerebral infarction caused by increased coagulation. In the field of ophthalmology, there have been reports of ocular motility disorders [2] and visual field defects [3-4], but to the best of our knowledge, there are few reports of visual acuity reduction as the initial symptom. We report a case where Trousseau's syndrome was diagnosed after an ophthalmological examination due to reduced visual acuity as the presenting symptom.

## Case presentation

**Figure 1:**
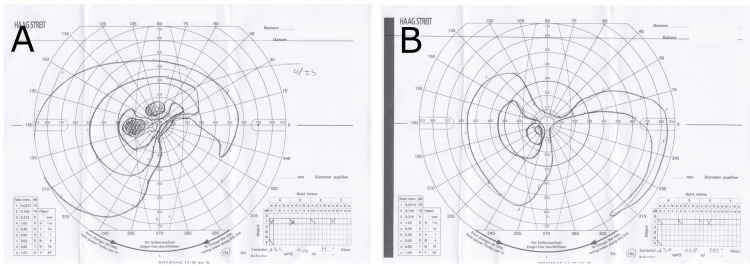
Goldmann visual field test A: Left eye; B: Right eye. Results: Both eyes have extensive visual field defects. A scotoma was observed in the superior and inferonasal directions in the right eye, and the superior and inferonasal regions in the left eye.

The patient is a 70-year-old male. The chief complaint is progressive vision loss starting several weeks ago. His medical history began in December 2021, when he stopped having bowel movements and could no longer eat, only drinking water. After eight days, he visited a primary care physician due to right-sided abdominal tenderness. A simple abdominal CT scan revealed multiple liver tumors, gallstones, and narrowing of the descending colon. A tumor shadow in the tail of the pancreas was also noted, and a contrast-enhanced CT scan the following day showed suspected pancreatic cancer, multiple enlarged periaortic lymph nodes, and a nodular shadow in the left upper lobe of the lung. Pancreatic cancer with multiple metastases (Stage IV) was suspected, and gemcitabine monotherapy was introduced. On the next day, a liver tumor biopsy identified pancreatic tissue, confirming the diagnosis of multiple metastases from pancreatic cancer. Chemotherapy for the second course started the following week. After two weeks, the patient began experiencing a subjective symptom of bilateral vision loss, and there has been no improvement in symptoms for a week. Then he was referred to a general comprehensive ophthalmologist. The patient's corrected visual acuity was significantly reduced, with the right eye (0.04) and left eye (0.08), and a Goldman visual field test (Figure 1) showed extensive visual field defects. Retinal microvascular damage was also suspected in both eyes, so he was referred to the Department of Ophthalmology at the University of Tokyo Hospital for further examination and treatment for cancer-related retinopathy.

The patient's medical history included type 2 diabetes, hypertension, hypothyroidism, and occlusive arteriosclerosis (ASO), among other conditions. He was taking glimepiride and ipragliflozin L-proline for type 2 diabetes, cilostazol and atorvastatin calcium hydrate for ASO, amlodipine besylate for hypertension, and levothyroxine sodium hydrate for hypothyroidism. There were no significant findings in his lifestyle history, habits, medications, allergies, or family history.

**Figure 2:**
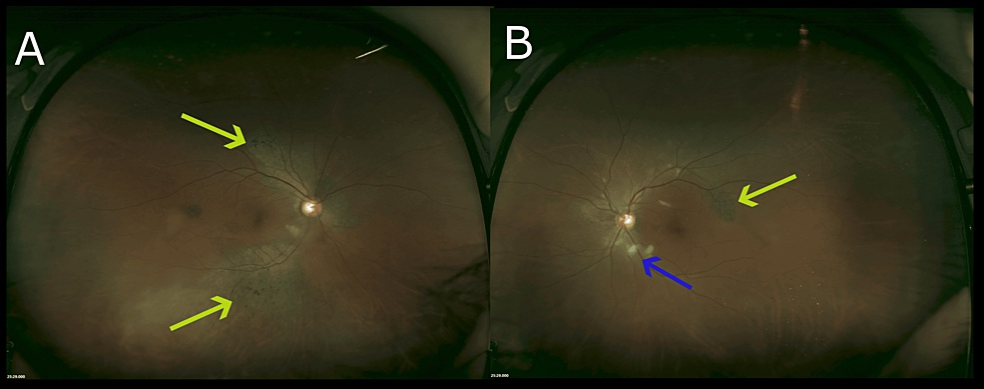
Optos ultra-widefield fundus imaging Both eyes show uneven retinochoroidal atrophy (yellow arrows) and scattered soft drusen (blue arrow) resembling retinal pigmentary degeneration in the arcades. No obvious inflammation, such as vitreous opacities, was confirmed.

**Figure 3:**
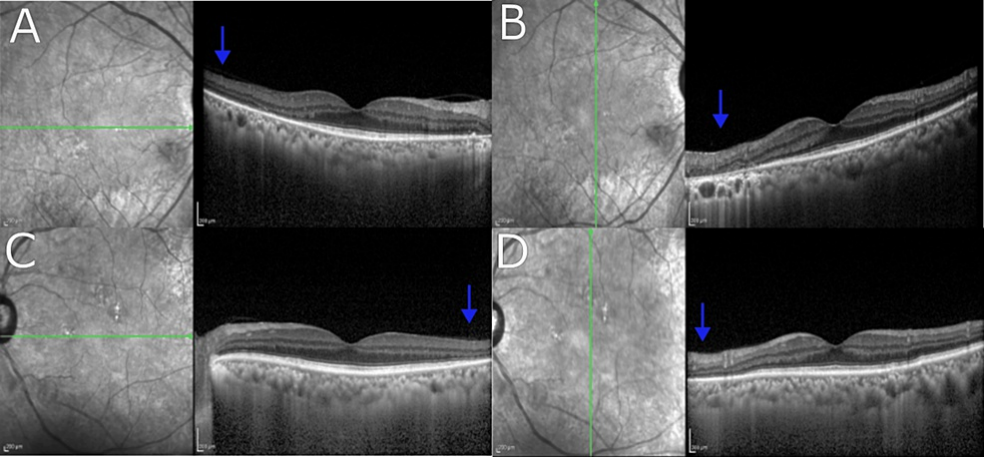
Optical coherence tomography (OCT) Results: No obvious abnormalities were observed in the macular region, but the disappearance of the ellipsoid zone (EZ, blue arrows) was observed in some areas outside the macular region.

At our clinic, the patient's corrected visual acuity was also reduced, with the right eye (0.04) and left eye (0.08). Pupillary light reflexes were normal bilaterally with no relative afferent pupillary defect (RAPD). There was no corneal opacity, deposits on the posterior corneal surface, or anterior segment inflammation. No significant abnormalities were found in the lens or vitreous. Ultra-wide-field fundus imaging with the Optos 200TX (Nikon) (Figure 2) was performed. Fundus findings included multiple localized areas of retinal and retinal pigment epithelial atrophy in both eyes, as well as scattered soft drusen around the optic nerve head. The optical coherence tomography (OCT) image captured by the Heidelberg SPECTRALIS (Figure 3) showed a loss of the ellipsoid zone (EZ) in some parts outside the macula. Autofluorescence fundus imaging with the Heidelberg retina angiograph 2 (HRA2) showed hypoautofluorescence in both eyes, corresponding to the areas of retinal atrophy.

**Figure 4:**
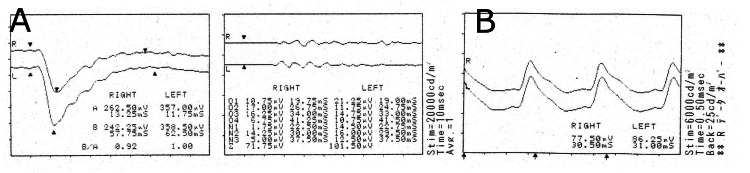
Electroretinogram (ERG) Findings: A mild reduction in the b-wave amplitude was observed in the photopic ERG. The flattening of the scotopic ERG typical of cancer-associated retinopathy (CAR) was not detected.

**Figure 5:**
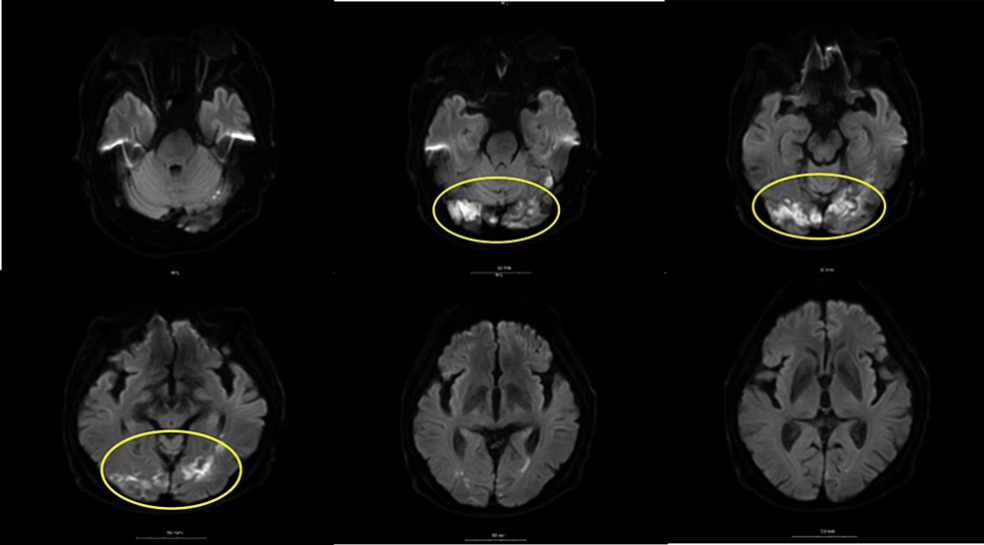
Magnetic Resonance Imaging of the brain Serial head MRI images were obtained at a local hospital for further examination. High signal findings were observed in both occipital lobes and cerebellar hemispheres in diffusion-weighted images (DWI). The MRI findings were diagnosed as subacute cerebral infarction.

The full-field electroretinogram (ERG) (Figure 4) showed only mild attenuation of b-wave amplitude in the cone ERG. Additionally, the anti-recoverin antibody test obtained on the day of consultation was negative. Due to the lack of significant macular and retinal abnormalities to explain the severe vision loss and the difficulty in differential diagnosis, a head MRI was performed for further examination to differentiate between the anterior and posterior visual pathways. The head MRI acquired in March 2022 (Figure 5), showed high signal findings in the bilateral occipital lobes and both cerebellar hemispheres in the diffusion-weighted image (DW1), leading to a diagnosis of subacute cerebral infarction, though the visual pathway other than the occipital lobe such as bilateral optic nerve, optic chiasm was unremarkable. On the same day, the patient was admitted to a primary care internal medicine department with a diagnosis of multiple cerebral infarctions, and further examination and treatment were pursued.

Blood tests revealed an elevated white blood cell count of 11,100 /μL, elevated fibrinogen at 529.0 mg/dL, elevated D-dimer at 5.5 μg/mL, and increased fibrinogen degradation products (FDP), leading to a diagnosis of Trousseau syndrome. Considering the time passed from the onset of the multiple cerebral infarctions, treatment with continuous low molecular weight heparin infusion was initiated. In subsequent contrast-enhanced CT scans, tumor enlargement was observed, and chemotherapy with Tegafur-gimeracil-oteracil potassium (TS-1) was administered. Following treatment, the patient received home care.

## Discussion

In this case, we observed Trousseau syndrome with vision loss in both eyes as the initial symptom. According to a review of 31 cases diagnosed with Trousseau syndrome by Lei et al. [5], there were reports of hemiplegia in 12 cases, dizziness in six cases, articulation disorder in five cases, motor dysfunction in four cases, facial nerve palsy in two cases, and aphasia in two cases as systemic symptoms. To our knowledge, there have been reports of Trousseau syndrome with ocular symptoms (Table 1), including two cases with visual field defects [1,2] and two cases with nystagmus [3,4]. However, all of these cases were accompanied by other systemic findings, such as aphasia, nausea, consciousness disorder, difficulty walking, and cerebellar ataxia.

**Table 1:** Reports of Trousseau syndrome Visual field defects in two cases and nystagmus in two cases have been reported, and the three cases had concurrent symptoms such as aphasia, nausea, consciousness disorders, difficulty walking, and cerebellar ataxia.

Reference	Primary Lesion	Ocular Symptoms	Systemic Symptoms
Suwa et al. [2]	Pancreatic cancer	Lower-left 1/4 blindness	None
Koishi et al. [3]	Rectal cancer	Lower-left visual field defect	Loss of consciousness & aphasia 2 days later
Yamazaki et al. [4]	Pharyngeal cancer	Nystagmus, diplopia	Nausea, difficulty walking
Kawase et al. [5]	Gastric cancer	Nystagmus	Right cerebellar ataxia, right facial nerve palsy
Current case	Pancreatic cancer	Decreased vision & visual field defect	Dizziness 1 month later

There have been reports of Trousseau syndrome cases with visual symptoms as the only initial presentation. Takeuchi et al. [6] reported a 23-year-old woman diagnosed with Trousseau syndrome associated with ovarian cancer after experiencing retinal artery occlusion in one eye. The patient had high signal findings in the cerebral cortex and basal ganglia on MRI, elevated D-dimer, and increased CA19-9 and CA125 levels in the blood test. Kunitake et al. [7] reported a 64-year-old woman diagnosed with Trousseau syndrome due to ovarian cancer after experiencing amaurosis fugax in both eyes alternately. Both cases had multiple cerebral infarctions following the onset of unilateral retinal artery occlusion. The patient had high signal findings in the cerebral cortex and basal ganglia on MRI, elevated D-dimer, and increased CA19-9 and CA125 levels in the blood test [7].

In summary, the present case illustrates Trousseau syndrome with vision loss as the initial symptom, which is a rare presentation. Careful evaluation, including neurological examination and imaging studies, is essential to diagnose and manage patients with Trousseau syndrome presenting with visual symptoms.

Cerebral infarctions can cause various symptoms, depending on the location of the lesion. In the present case, lesions occurred in both occipital lobes and the cerebellar hemispheres, which could have resulted in symptoms such as difficulty walking and cerebellar ataxia. Generally, bilateral occipital lobe infarctions are thought to primarily cause visual field defects such as homonymous hemianopia. Although central vision is often preserved in cases with limited lesion extent due to macular sparing, this case presented with vision loss, deviating from the typical pattern of cerebral infarction. Further neurological evaluation is warranted for this case.

In this case, the differential diagnoses included cancer-associated retinopathy, retinitis pigmentosa, and posterior uveitis, based on fundus findings and the subacute progression. Central retinal artery occlusion typically presents with acute onset, but there were no hyperintense lesions in the inner retinal layers of the macula, and the sudden onset in both eyes is considered rare. However, some retinal lesions were accompanied by soft exudates, suggesting the possibility of local retinal ischemia.

Further detailed OCT imaging would be preferable to assess the possibility of retinal ischemia. In this case, the patient's overall condition was poor, and the discovery of cerebral infarctions made their treatment a priority, preventing the performance of fluorescein angiography. Thus, it is difficult to completely rule out retinal ischemia. As for cancer-associated retinopathy, the possibility is considered low due to the limited reduction in amplitude on the electroretinogram (ERG), the absence of ERG flattening, and the negative anti-recoverin antibody result.

Similarly, retinitis pigmentosa is considered unlikely because the ERG findings do not match, and characteristic fundus findings such as bone spicules are not observed.

While posterior uveitis is also a possible consideration, no significant inflammatory findings such as vitreous cells were observed in the anterior or posterior segments of the fundus. However, fluorescein angiography was not performed, so it is difficult to rule out the possibility of vasculitis.

This case is unique in that multiple differential diagnoses were considered as the cause of visual acuity decline due to the fundus and OCT abnormalities present at the initial examination. After various tests, other diagnoses were excluded, and Trousseau syndrome was ultimately determined. However, in cancer patients like this case, the possibility of other differential diagnoses should also be considered. It is important to include Trousseau syndrome in the differential diagnoses while also performing appropriate imaging tests to rule out other pathologies.

In Trousseau syndrome, blood test findings can be used as criteria for diagnosis, including increased D-dimer and fibrinogen levels due to coagulation abnormalities, elevated white blood cell count and c-reactive protein (CRP), low albumin and total cholesterol levels due to malnutrition, and increased blood urea nitrogen (BUN) due to dehydration. In this case, the blood tests performed on the same day as the brain MRI revealed elevated white blood cell count, fibrinogen, and D-dimer levels, suggesting a hypercoagulable state. Other blood test findings indicated hypoalbuminemia and decreased kidney function. These findings were similar to those reported by Nogawa and colleagues [8].

The diagnosis of Trousseau syndrome is based on the DIC diagnostic criteria (DIC score) established in 1988 [9]. The criteria for DIC diagnosis include increased FDP, decreased platelets, decreased fibrinogen, and prolonged prothrombin time. These factors are scored, and a score of seven or higher indicates DIC. If the score is six or higher but does not reach seven, supplementary tests such as D-dimer may be used.

However, Nogawa et al. reported [8], that only 26 out of 74 cases with suspected Trousseau syndrome met the diagnostic criteria for DIC, accounting for 35.1% of the cases, which is not high. On the other hand, even in cases that do not meet the above DIC diagnostic criteria, an increase in D-dimer and FDP levels is considered useful for diagnosis. Therefore, in cases of embolic cerebral infarction or thrombosis with DIC, or cases with a DIC score of less than seven but an increase in D-dimer and FDP levels, a thorough investigation for malignant tumors should be considered after excluding infections, congenital coagulation abnormalities, primary antiphospholipid syndrome, vascular malformations, and severe vascular stenosis.

Regarding treatment, continuous infusion of low molecular weight heparin has been reported to be effective [8], and in this case, treatment was performed with continuous infusion of low molecular weight heparin.

In the field of ophthalmology, even in cases of retinal artery occlusion due to increased coagulation, such as elevated D-dimer and FDP levels, the possibility of hidden adenocarcinoma cannot be ruled out even if the DIC score is not met. For example, adenocarcinomas, ovarian cancer in young women, lung cancer in elderly men, and pancreatic cancer, as in this case, can be considered. When arterial occlusion and other conditions are confirmed in patients with ophthalmologic symptoms, it is important to obtain a thorough history, including past medical history and current treatments, during the interview. However, if D-dimer and FDP levels are elevated in blood tests, the possibility of hidden adenocarcinoma cannot be ruled out. Therefore, it may be worth considering the addition of adenocarcinoma blood markers such as carcinoembryonic antigen (CEA) and CA19-9 that can be performed in the same blood test.

## Conclusions

In conclusion, We present a case of a patient who experienced bilateral vision loss and was subsequently diagnosed with Trousseau's syndrome associated with pancreatic cancer. Elevated coagulability markers, including D-dimer, efficiently led to the diagnosis. Reports of Trousseau syndrome with vision loss as the initial symptom are scarce, as far as our literature search revealed. When diagnosing vision loss and visual field abnormalities in patients with cancer, it is important to consider the possibility of this syndrome and to evaluate imaging and coagulation abnormalities.
